# Metagenomic Approaches to Explore the Quorum Sensing-Mediated Interactions Between Algae and Bacteria in Sequence Membrane Photo-Bioreactors

**DOI:** 10.3389/fbioe.2022.851376

**Published:** 2022-04-05

**Authors:** Xiaogang Wu, Lingrui Kong, Juejun Pan, Yiming Feng, Sitong Liu

**Affiliations:** ^1^ College of Environmental Sciences and Engineering, Peking University, Beijing, China; ^2^ Key Laboratory of Water and Sediment Sciences, Ministry of Education, Peking University, Beijing, China

**Keywords:** metagenomic, nitrogen removal, signaling, algal–bacterial interaction, indole-3-acetic acid

## Abstract

Algal–bacterial water treatment is more effective for better harvesting and promotes energy savings than other traditional treatments, while the relationships between them are multifarious. Among all the interactions, quorum sensing plays an essential ecological role. However, the relative contributions of signaling in the interaction between algae and bacteria are not clear. To elucidate the role of quorum sensing by indole-3-acetic acid (IAA) in terms of the algal–bacterial interaction during the nitrogen removal process, the bioreactors, respectively, inoculated with *Chlorella*, *Phormidium,* and both of them were started. We manifest the existence of multiple signaling-related proteins by alignment with the constructed database, and the signaling was analyzed using metagenomic sequence data obtained during bioreactor operation. We found that IAA was mainly synthetized depending on indole-3-acetamide (IAM) and indole-3-pyruvic acid (IPA) pathways by calculating the gene abundance of IAA synthetase. Both *Chlorella* and the co-culture reactor possessed higher nitrogen removal rate (NRR) than the *Phormidium* reactor, and the abundance profile of the signaling-related gene is similar with the NRR. The signaling-related gene abundance increased in *Chlorella* and co-culture reactors but decreased in the *Phormidium* reactor. *Pseudomonas*, *Hydrogenophaga,* and *Zoogloea* are the dominant signaled bacteria. *Chlorella* is the dominant signaled algae. The relative abundance of total signaled bacteria in the whole bacterial community increased during the start-up in *Chlorella* and co-culture reactors. According to the network analysis, phytoplankton prefers to positively correlate with signaled bacteria than non-signaled bacteria, which indicated that the signaling influences the algal–bacterial interaction. These findings hint at the significance of algal–bacterial signaling in this interkingdom interaction during nitrogen removal.

## Introduction

Algae-based wastewater treatment has already been extensively used for nitrogen removal (Abeysiriwardana-Arachchige et al., 2020). It has been found that the algal–bacterial interaction influences wastewater treatment. Nevertheless, the interaction between algae and bacteria is still elusive during the water treatment using algae (Ramanan et al., 2016). Treatment systems of algal–bacterial interaction have been widely used for wastewaters with rich nutrients since the 1950s. It was already been proved that bacteria help in the flocculation of algae and would be effective for better harvesting of the algal and bacterial biomass, also reducing the costs (Kim et al., 2014; Lee et al., 2013). Additionally, to effectively treat the dissolved methane in anaerobically treated wastewaters, the synergism between algae and methane-oxidizing bacteria was used, which are otherwise released into the atmosphere in other processes based on aeration (van der Ha et al., 2012). Considering the relatively minuscule utilization of energy, treatment efficiency, and biomass production which could be valorized, the algal–bacterial water treatment process could be the main alternative technology to aeration-based technologies such as activated sludge treatment (Kim et al., 2014; Ramanan et al., 2016). The conservative estimate shows that treatment with the algal–bacterial process can trigger 100-fold energy savings than traditional activated sludge treatment (Kang et al., 2015). Nevertheless, the attached systems with biofilms are more sophisticated compared to the systems of suspended culture, as systematic investigations on the fundamental processes are less understood, and studies on the role of algal–bacterial interactions, especially in the engineered membrane systems for treating sewage, are sparse (Kesaano and Sims, 2014).

Increasing attention has been paid to the interaction between algae and bacteria. It is the fundamental ecological relationship in water environments by regulating biomass production and nutrient cycling at the base of the aquatic food web (Seymour et al., 2017). Algal–bacterial interactions are diverse and often highly complex, from cooperative to competitive (Amin et al., 2012; Durham et al., 2015). The relationship between algae and bacteria is based on resource provision, and it can be either exploitative or reciprocal in an aquatic environment at the simplest level (Cole, 1982). Heterotrophic bacteria in a water environment obtain a large fraction of their carbon demand directly from phytoplankton. More than 50% of the carbon fixed by algae is ultimately consumed by heterotrophic bacteria (Fouilland et al., 2014; Fuhrman and Azam, 1982). Algal cells release large quantities of dissolved and labile organic carbon into the surrounding water, and bacteria derive organic material from algae primarily depending on assimilation (Biddanda and Pomeroy, 1988; Larsson and Hagstrom, 1979; Piontek et al., 2011; Teeling et al., 2012). From the perspective of an algal cell, bacteria can be competitors for inorganic nutrients, but they also provide the limiting macronutrients to algae by remineralization (Joint et al., 2002; Legendre and Rassoulzadegan, 1995). When the nutrient supply is limited, algal growth is particularly benefited from the bacterial delivery of the generated phosphorus and nitrogen (Amin et al., 2009; Croft et al., 2005; Falkowski, 1994). According to previous investigations, algae-associated bacterial communities mainly contain members of Alteromonadaceae, Flavobacteraceae, and Roseobacter clade (Amin et al., 2012; Buchan et al., 2014; Goecke et al., 2013; Green et al., 2015; Guannel et al., 2011; Rooney-Varga et al., 2005; Teeling et al., 2012; van Tol et al., 2017; Zhou et al., 2020). Additionally, algal–bacterial interactions involve the exchange of multifarious substances such as growth resources, nutrients, and infochemicals. The metabolism and sensing of these chemical currencies underpin the interaction between the algae and bacteria, spanning competition, antagonism, commensalism, and obligate mutualism (Mayali and Azam, 2004; Windler et al., 2014; Seymour et al., 2017).

Among all the interactions between algae and bacteria, interkingdom signaling is the language *via* chemical signals and plays an ecological role (Zhou et al., 2016; Cirri and Pohnert, 2019). The bacterial consortium associated with a globally distributed diatom has already been unraveled (Amin et al., 2015). The result showed that cell division of eukaryotic phytoplankton was promoted *via* the exchange of the auxin indole-3-acetic acid (IAA) from *Sulfitobacter*. The algal cyclin was regulated by bacterial IAA during the quorum sensing. Thereby, the results verified that the algal–bacterial interaction is regulated by the production and secretion of infochemicals. Generally, the bacterial synthesis of IAA contains three different pathways; they are, respectively, the indole-3-acetamide (IAM) pathway, indole-3-pyruvic acid (IPA) pathway, and indole-acetaldoxime/indole-3-acetonitrile (IAOx/IAN) pathway (Spaepen et al., 2007; Duca et al., 2014; Amin et al., 2015). In the IAM pathway, indoleacetamide hydrolase is the key enzyme which catalyzed the last step from indoleacetamide to IAA. In the IPA pathway, indoleacetaldehyde dehydrogenase is the key enzyme which catalyzed the last step from indoleacetaldehyde to IAA. In the IAOx/IAN pathway, indoleacetonitrile nitrilase is the key enzyme which catalyzed the last step from indoleacetonitrile to IAA. However, the role of interkingdom signaling in the algal–bacterial interaction needs to be more investigated, and the relative importance of quorum sensing is not clearly elucidated.

To explore the quorum sensing between phytoplankton and bacteria during the nitrogen removal process, bioreactors, respectively, inoculated with *Chlorella*, *Phormidium,* and both of them were started. The abundance of IAA-related proteins and the community structure of signaled bacteria and algae are analyzed by metagenomic sequencing. Additionally, the algal–bacterial interactions are also evaluated by a co-occurrence network. The results offer deeper insights into the signaling between phytoplankton and bacteria during the photo-bioreactor start-up process and hint at the relative importance of algal–bacterial signaling in the interactions.

## Materials and Methods

### Bioreactor Operation

Three sequence membrane photo-bioreactors (SMPBRs) were started up for the experiment; the first two reactors were inoculated with single algae *Chlorella* and *Phormidium*, and the other reactor was employed as a co-culture for the two algae with inoculation ratios of 1:1 according to dry weight. The initial biomass concentration in the three reactors was adjusted to 200 mg/L prior to the start of the experiments. The microalgae strains *Chlorella* sp. (FACHB-31) and *Phordium* sp. (FACHB-1129) were purchased from the Freshwater Algae Culture Collection at the Institute of Hydrobiology, Wuhan, China. The experimental conditions for the continuous operation of different phases are shown in Table 1. To evaluate the nitrogen removal rate (NRR) during the start-up, ammonium (NH_4_^+^ **-N**), nitrite (NO_2_^−^ **-N**), and nitrate (NO_3_^−^ **-N**) were determined according to the APHA Standard Methods (APHA, 2005).

**TABLE 1:** Experimental conditions for the continuous operation of the SMPBR.

Phase	Day	Influent NH_4_^+^-N (mg/L)	Influent COD (mg/L)
1	0–14	30–65	180 ± 5
2	14–42	60 ± 5	180 ± 5
3	42–70	70 ± 5	70 ± 5

The sequence membrane photo-bioreactors (SMPBR) had a working volume of 3 L with dimensions of 0.16 m, 0.16 m, and 0.2 m (length, width, and height, respectively). A hollow fiber membrane module made of polyvinylidene fluoride (PVDF) with a nominal pore size of 0.45 μm, and a total effective filtration area of 0.1 m^2^, was directly submerged in the bioreactor. The magnetic stir was employed at 300 rpm to homogenize the liquid and avoid biofilm formation. An LED light was twined around the surface of the reactor, and a fixed 12 h dark/12 h light cycle was applied on a daily basis with a light intensity of 6,000 lux at the surface of the algal mixed liquor during light exposure. The pH in the reactor was controlled in the range of 7.5 ± 0.3 by NaOH and HCl solution. All the reactors were operated under a batch and semi-continuous mode, and the SMPBR cycle consists of the following phases: feeding (30 min), mixing (23 h), and withdrawal and filtration (30 min). The algal biomass was retained by the membrane and was weekly harvested. One-third of the mixed liquor (1 L) was directly discharged every 7 days, in a biomass retention time (BRT) of 21 days.

The reactors were fed with synthetic low C/N ratio wastewater and were operated in four phases. In phase I (acclimatization phase, days 1–14), the ammonium nitrogen was gradually increased from 30 mg/L to 60 mg/L to acclimate the algae, and the COD was 180 ± 5 mg/L. In phase II (days 15–42), the concentration of NH_4_^+^-N and COD was maintained at 60 ± 5 and 180 ± 5 mg/L, respectively. In phase III (days 43–70), the C/N ratio was decreased from 3:1 to 1:1, and the concentrations of NH_4_^+^-N and COD were both kept at 70 ± 5 mg/L. Other contents of the synergetic wastewater were constant in all phases, with 6.5 ± 0.5 mg/L total phosphorus (TP), in the form of PO_4_^−^-P, 60 mg/L NaHCO_3_, 20 mg/L MgSO_4_, 10 mg/L CaCl_2_, and 1 ml trace solution.

### Metagenomic Sequencing

The biological samples were collected after 42 and 70 days, and 200 mg of sample in each reactor was centrifuged at 8,000 rpm for 5 min. The DNA extraction and metagenome sequencing were conducted as described in the supplemental material. The clean reads were classified using Kaiju to explore the microbiota compositions. The Kaiju was run against the nr_euk database (built in May 2020) using default parameters (Menzel et al., 2016).

### Dataset Construction

The procedure for the construction of the sequence datasets related to IAA is performed as previously described in the construction for the bacterial communication gene (Tang et al., 2018). To explore the signaled bacterial community in a bioreactor culture, three identified proteins related to IAA synthetase of bacteria were selected (Amin et al., 2015; Duca et al., 2014; Spaepen et al., 2007). They are the last-step enzymes in three different pathways: indoleacetamide hydrolase, indoleacetaldehyde dehydrogenase, and indoleacetonitrile nitrilase. Furthermore, the algal cyclin regulated by IAA was also selected (Amin et al., 2015). We retrieved all available sequences from a non-redundant (November 2021) database using the NCBI search tool and Swiss-Prot of the UniProt database (November 2021). Each subtype of IAA and cyclin (Supplementary Tables S1, S2) was examined to obtain a clean version of the datasets by checking annotation, discarding redundant and partial sequences (Fang et al., 2014). Non-redundant sequences were picked by cd-hit at a 97% similarity (Li and Godzik, 2006; Yang et al., 2016).

### Screening for Functional Genes in Reactors

The detection of bacterial IAA synthetase and algal cyclin from the metagenomic sequencing data of all samples was conducted by sequence homology alignments using the BLASTX algorithm with an E-value ≤10^–5^. If the best hit of a BCG read had >50% sequence identity and the alignment length was >25 amino acids, the read was annotated as an IAA synthetase-like read and algal cyclin-like read. To avoid bias, the abundance of bacterial IAA synthetase and algal cyclin was calculated by normalizing the reference sequence length. Therefore, the abundance of bacterial IAA synthetase and algal cyclin was calculated using the method of Reads Per Kilobase per Million mapped reads (RPKM) as previously described (Tang et al., 2018).

### Taxonomic Annotation

To identify the bacterial hosts of the IAA synthetase-like reads and algal hosts of cyclin reads, all of the aligned reads were searched against the NCBI NR database using BLASTX algorithm. The top ten hits with E-values≤1 × 10^–5^ were imported to MEGAN (MEtaGenome ANalyzer, Version 6). Then, MEGAN was used to explore the taxonomical origin of the dataset, employing the NCBI taxonomy (January 2021) to summarize the results. The lowest common ancestor (LCA) parameters were set at a min score of 50, top percent of 10.0, and min support of 0.01 (Huson et al., 2016; Huson and Weber, 2013). Potentially missed assignments were manually corrected. The reads were only classified at the genus level to ensure accuracy.

### Network Analysis Between Bacteria and Phytoplankton

To analyze the interaction between bacteria and phytoplankton, Spearman’s correlation coefficients were calculated. Correlations with ρ > 0.6 and *p* value < 0.01 were considered significant. In addition, the network was conducted and analyzed by Cytoscape (Shannon et al., 2003) to visualize the significant correlations between bacteria and phytoplankton.

## Results

### Nitrogen Removal Rates of Different Reactors

**FIGURE 1:**
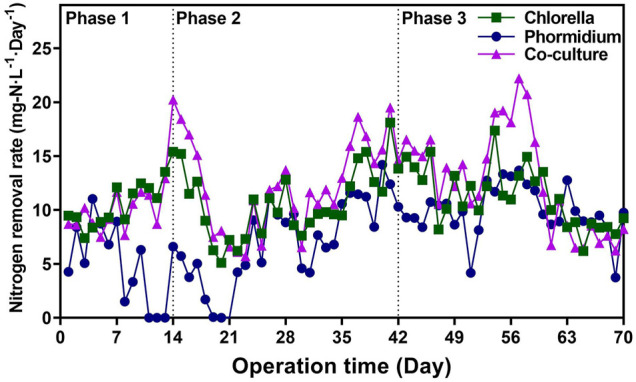
Nitrogen removal rates of different reactors in three phases.

Both *Chlorella* and co-culture reactors possessed higher nitrogen removal rate (NRR) value than the *Phormidium* reactor (Figure 1) During the whole period of the start-up, the NRR value of the *Chlorella* reactor was 10.85 ± 2.80 mg-N·L^−1^·d^−1^, the NRR value of the *Phormidium* reactor was 7.82 ± 3.80 mg-N·L^−1^·d^−1^, and the NRR value of the co-culture reactor was 12.05 ± 4.25 mg-N·L^−1^·d^−1^. In three different phases, the NRR values of *Chlorella* and co-culture reactors are invariably significantly higher than those of the other *Phormidium* reactor, respectively (t test, *p* value <0.05).

### Gene Abundance of Different IAA Synthetase Subtypes

**FIGURE 2:**
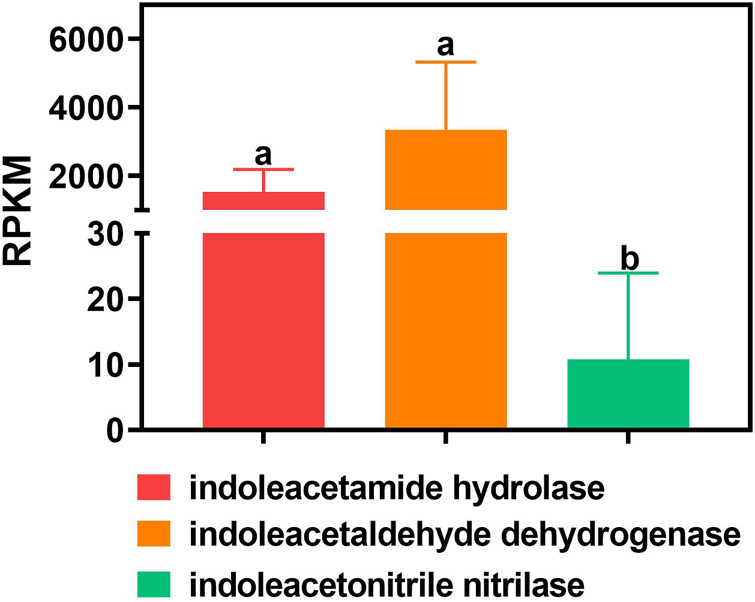
Gene abundance of different bacterial IAA synthetases in all samples. One-way ANOVA was used to analyze variations among all the treatments. Different small letters indicate significant differences at the *p* < 0.05 level.

The RPKM values of indoleacetamide hydrolase and indoleacetaldehyde dehydrogenase are significantly higher than those of indoleacetonitrile nitrilase in all samples (*p* value <0.05). In detail, the gene abundance of indoleacetamide hydrolase is 1,540.824 ± 651.879 RPKM in all the samples. The gene abundance of indoleacetaldehyde dehydrogenase is 3,350.513 ± 1976.065 RPKM in all the samples. Especially, the gene abundance of indoleacetonitrile nitrilase is merely 10.760 ± 13.169 RPKM in all the samples (Figure 2).

### Change Trends and Profiles of IAA-Related Protein in the Photo-Bioreactors

**FIGURE 3:**
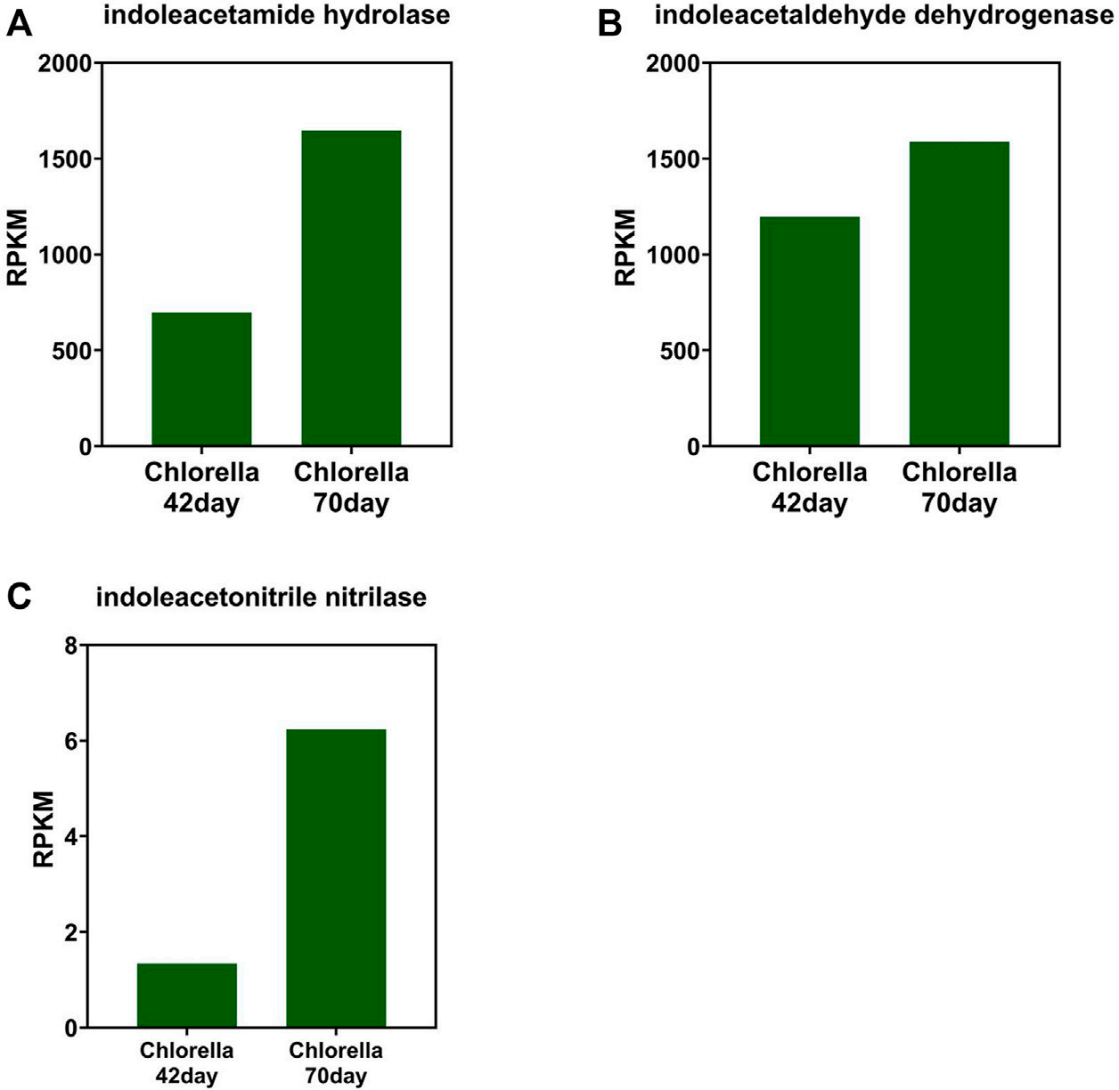
Gene abundance of bacterial IAA synthetase in the *Chlorella* reactor. **(A)** Indoleacetamide hydrolase, **(B)** indoleacetaldehyde dehydrogenase, and **(C)** indoleacetonitrile nitrilase.

**FIGURE 4:**
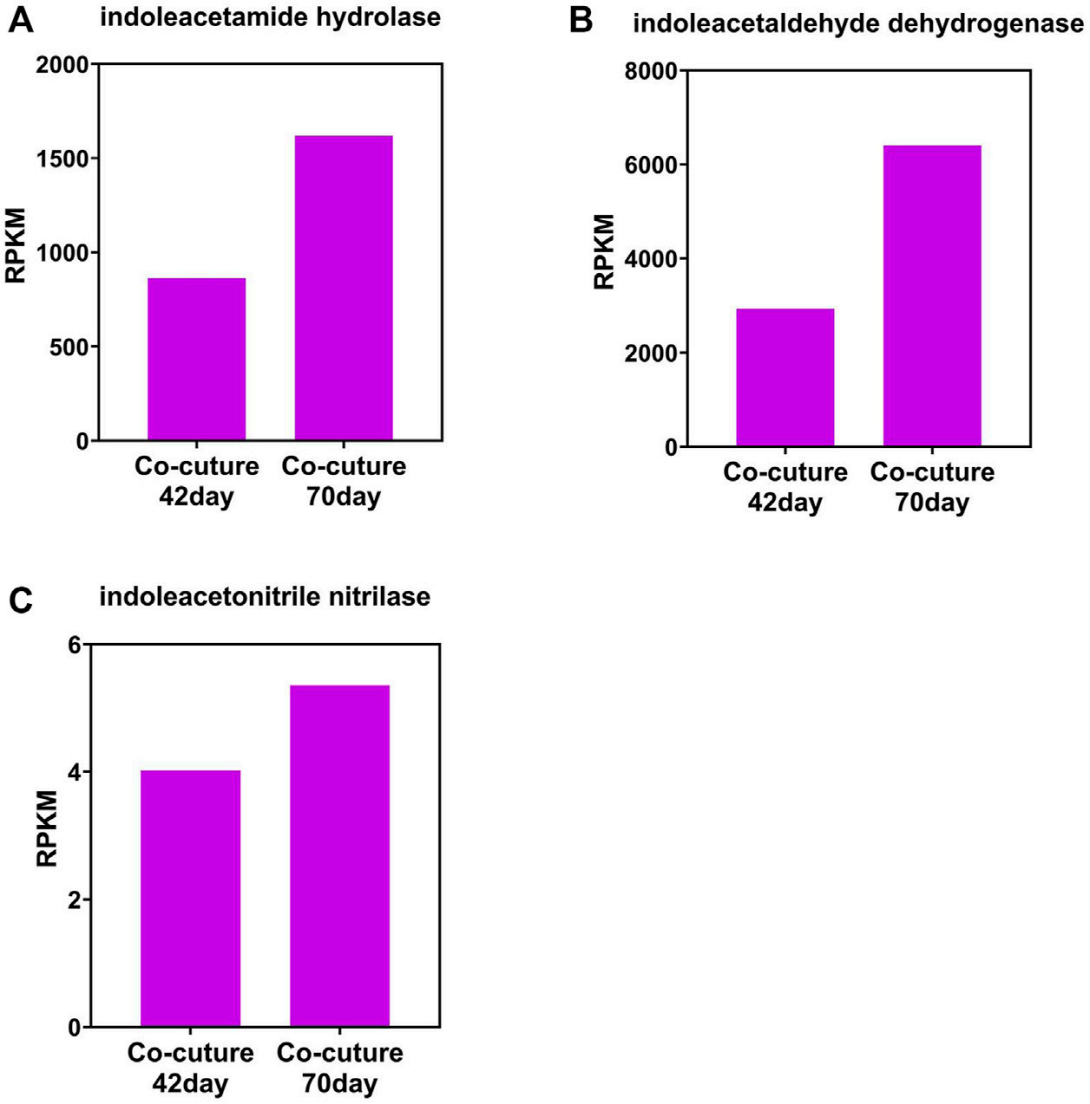
Gene abundance of bacterial IAA synthetase in the co-culture reactor. **(A)** Indoleacetamide hydrolase, **(B)** indoleacetaldehyde dehydrogenase, and **(C)** indoleacetonitrile nitrilase.

**FIGURE 5:**
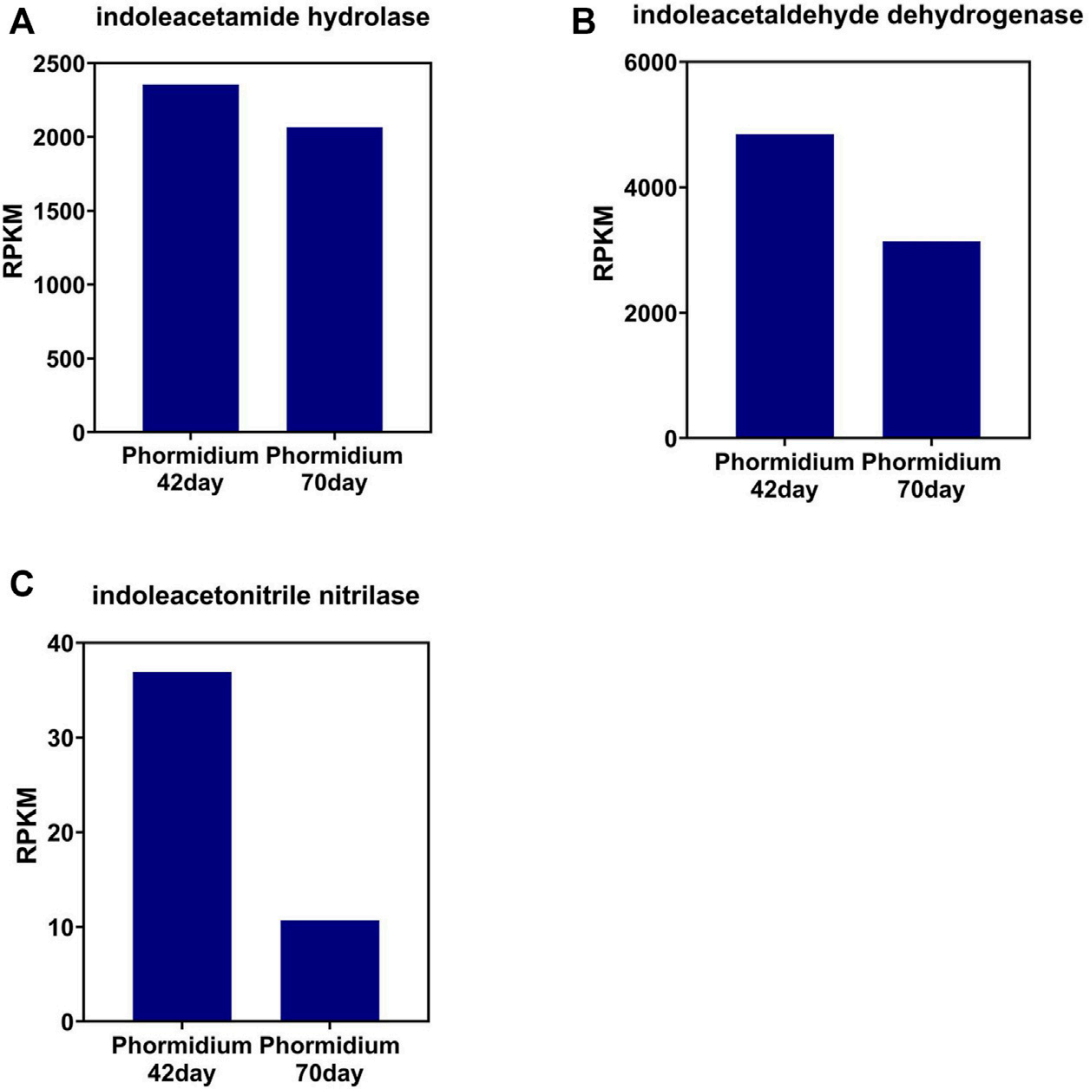
Gene abundance of bacterial IAA synthetase in the *Phormidium* reactor. **(A)** Indoleacetamide hydrolase, **(B)** indoleacetaldehyde dehydrogenase, and **(C)** indoleacetonitrile nitrilase.

The change tendency of IAA synthetase is different between that of the photo-bioreactor inoculated with eukaryotic *Chlorella* and prokaryotic *Phormidium*. In the *Chlorella* and co-culture reactors, the gene abundance of indoleacetamide hydrolase, indoleacetaldehyde dehydrogenase, and indoleacetonitrile nitrilase all increased from day 42 to day 70 during the start-up (Figures 3, 4). In the *Phormidium* reactor, the gene abundance of these IAA synthetases all decreased from day 42 to day 70 during the start-up (Figure 5).

**FIGURE 6:**
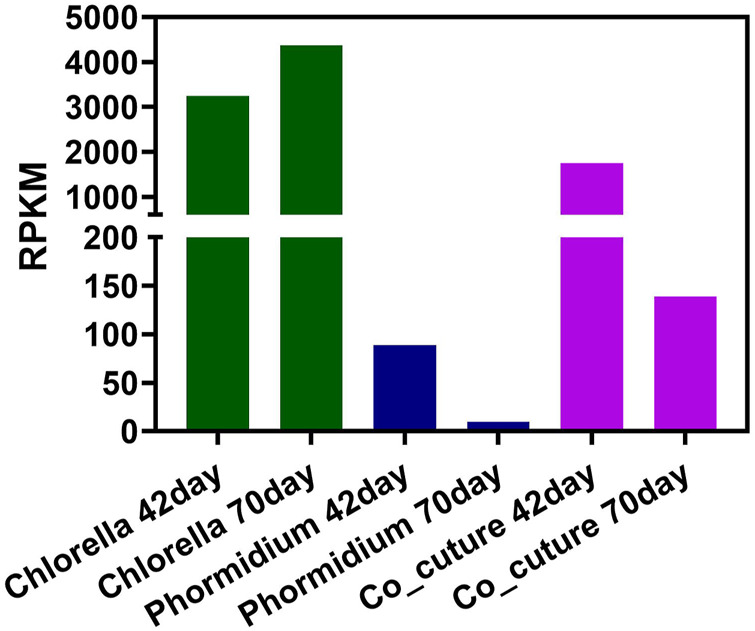
Gene abundance of algal cyclin in all samples.

The change tendency of cyclin is also different in that of the three kinds of reactors. In the *Chlorella* reactor, the gene abundance of cyclin increased from day 42 to day 70 during the start-up (Figure 6). In the *Phormidium* and co-culture reactors, the gene abundance of cyclin decreased from day 42 to day 70 during the start-up. Additionally, the abundance of algal cyclin in *Chlorella* and co-culture reactors is higher than that of the *Phormidium* reactor. In detail, the gene abundance of cyclin in the *Chlorella* reactor is 3,241.20 RPKM and 4,369.73 RPKM (day 42 and day 70, respectively). The gene abundance of cyclin in the co-culture reactor is 1752.07 RPKM and 138.84 RPKM (day 42 and day 70). Nevertheless, the gene abundance of cyclin in the *Phormidium* reactor is merely 88.73 and 9.77 (day 42 and day 70).

### Community Structure of the Signaled Bacteria and Phytoplankton

**FIGURE 7:**
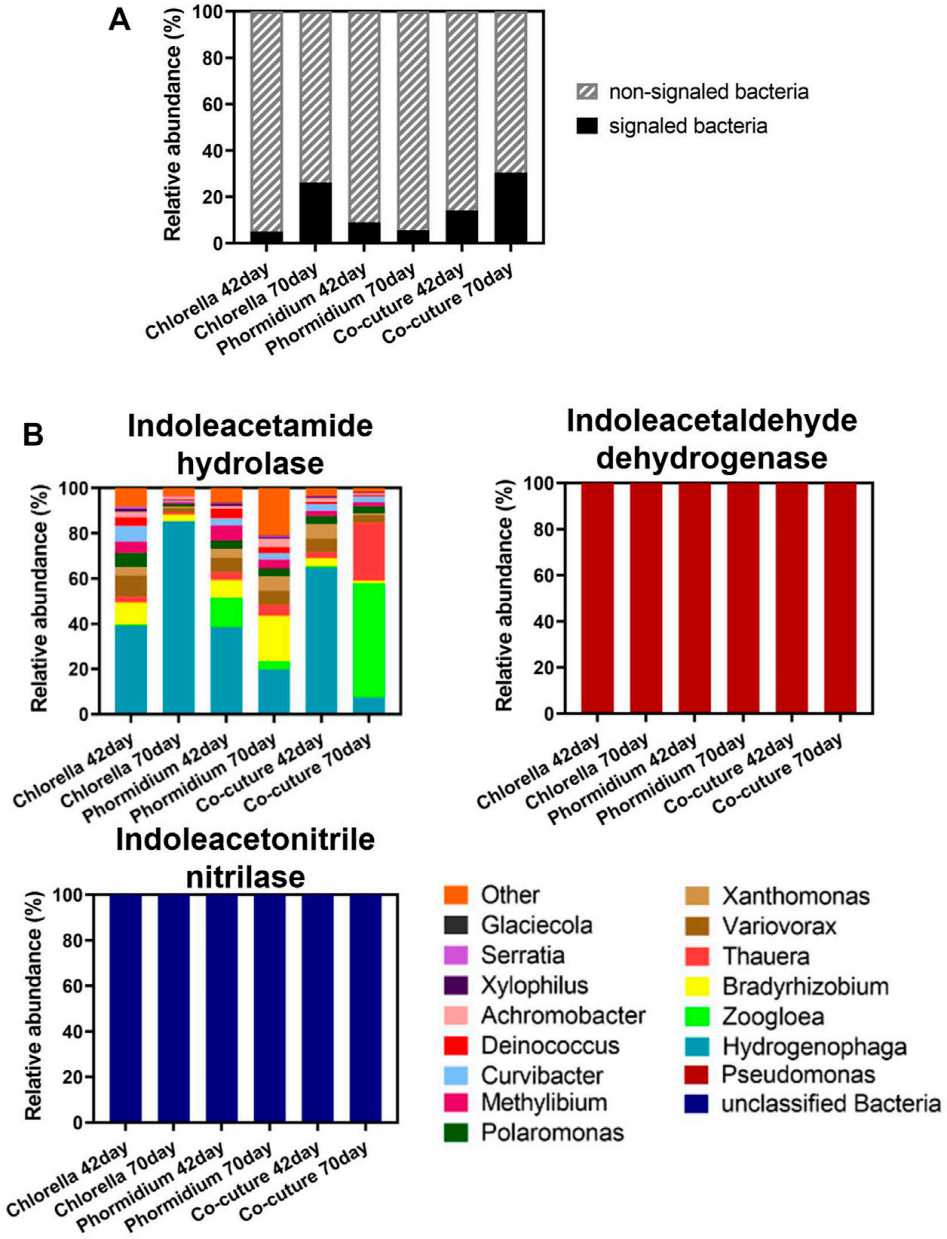
Community structure of signaled bacteria. **(A)** Subcommunity of signaled bacteria carrying IAA synthetase. **(B)** The relative abundance of signaled bacteria carrying IAA synthetase in the bacterial community.

The bacteria carrying IAA synthetase are the signaled bacteria in the different algal reactors (Figure 7). The relative abundance of the total signaled bacteria in the whole bacterial community increased during the start-up in *Chlorella* and co-culture reactors but decreased in the *Phormidium* reactor (Figure 7A). *Hydrogenophaga*, *Zoogloea*, *Bradyrhizobium,* and *Thauera* are the dominant signaled bacteria carrying indoleacetamide hydrolase. *Pseudomonas* is the only signaled bacterium carrying indoleacetaldehyde dehydrogenase in different reactors. Nevertheless, the bacteria carrying indoleacetonitrile nitrilase are unclassified in all reactors (Figure 7B).

**FIGURE 8:**
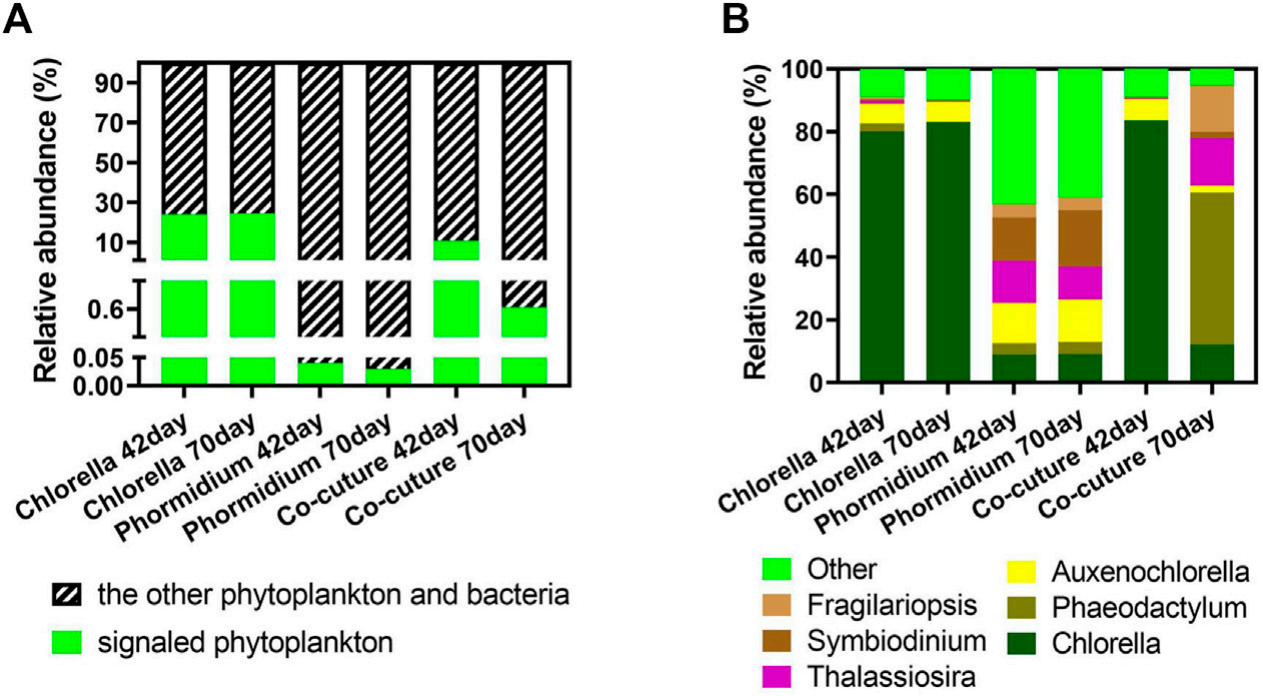
Community structure of signaled phytoplankton. **(A)** Subcommunity of signaled phytoplankton carrying cyclin. **(B)** The relative abundance of signaled phytoplankton carrying cyclin in the whole community.

The algae carrying cyclin protein are the signaled algae in the different algal reactors (Figure 8). The signaled phytoplankton possess high abundance in *Chlorella* reactors and co-culture reactors in the total community (algae and bacteria), and *Chlorella* is the dominant signaled phytoplankton carrying cyclin in these samples. Conversely, the signaled algae possess an extremely low abundance in the *Phormidium* reactor in the total community (algae and bacteria). *Phaeodactylum*, *Auxenochlorella*, *Thalassiosira*, *Symbiodinium,* and *Fragilariopsisare* are the indigenous algal genera from influent water.

### Co-Occurrence Network Between Bacteria and Phytoplankton

**FIGURE 9:**
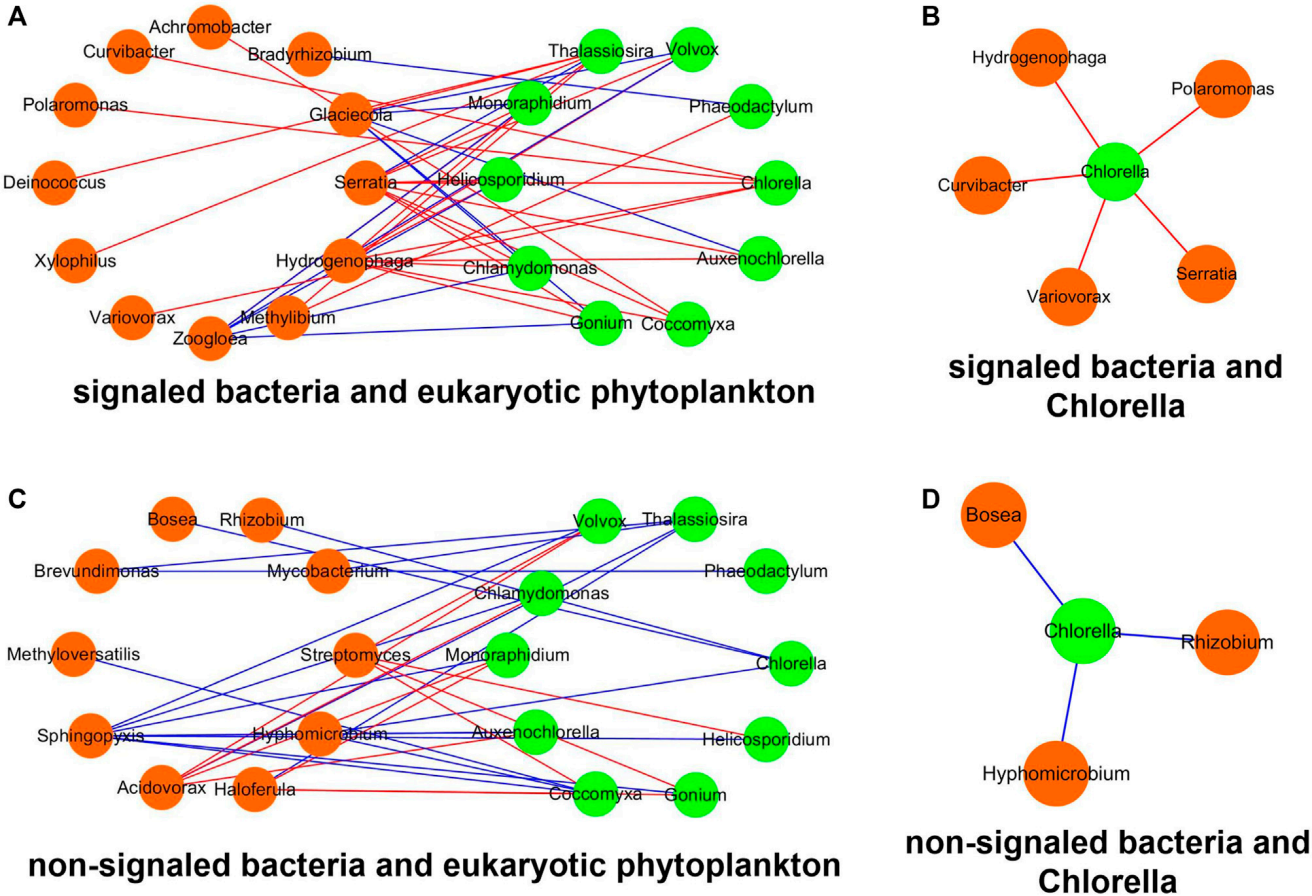
Network between bacteria and phytoplankton. **(A)** Signaled bacteria and eukaryotic phytoplankton, **(B)** non-signaled bacteria and eukaryotic phytoplankton, **(C)** signaled bacteria and *Chlorella*, and **(D)** non- signaled bacteria and *Chlorella*. Orange nodes represent bacteria, and green nodes represent phytoplankton. Red lines represent positive correlation, and blue lines represent negative correlation.

Since the bacterial IAA only influences the reactors inoculated with eukaryotic *Chlorella*, the network analysis was performed to elucidate the correlation between bacteria and eukaryotic phytoplankton (Figure 9). The density, heterogeneity, and centralization value of the network between signaled bacteria and phytoplankton are higher than those of the network between non-signaled bacteria and phytoplankton (Table 2). The significant correlations between signaled bacteria and *Chlorella* are all positive, and the signaled bacteria contain *Serratia*, *Hydrogenophaga*, *Polaromonas*, *Curvibactera,* and *Variovorax*. The significant correlations between non-signaled bacteria and *Chlorella* are all negative, and the non-signaled bacteria contain *Bosea*, *Rhizobium,* and *Hyphomicrobium*.

**TABLE 2:** Characteristics of networks between bacteria and eukaryotic phytoplankton.

Network index^a^	Signaled bacteria and eukaryotic phytoplankton	Non-signaled bacteria and eukaryotic phytoplankton
Network density	0.165	0.158
Network heterogeneity	0.680	0.494
Network centralization	0.290	0.234
Information centrality	1.893 + 0.320^b^	1.869 ± 0.439
Closeness centrality	0.384 ± 0.081	0.357 ± 0.071

a Network density, heterogeneity, and centralization represent characteristics of the whole network. Information and closeness centrality represent characteristics of each node in the network.

b Values represent mean ± SD.

### The Relative Importance of Algal–Bacterial Signaling in the Interaction

**FIGURE 10:**
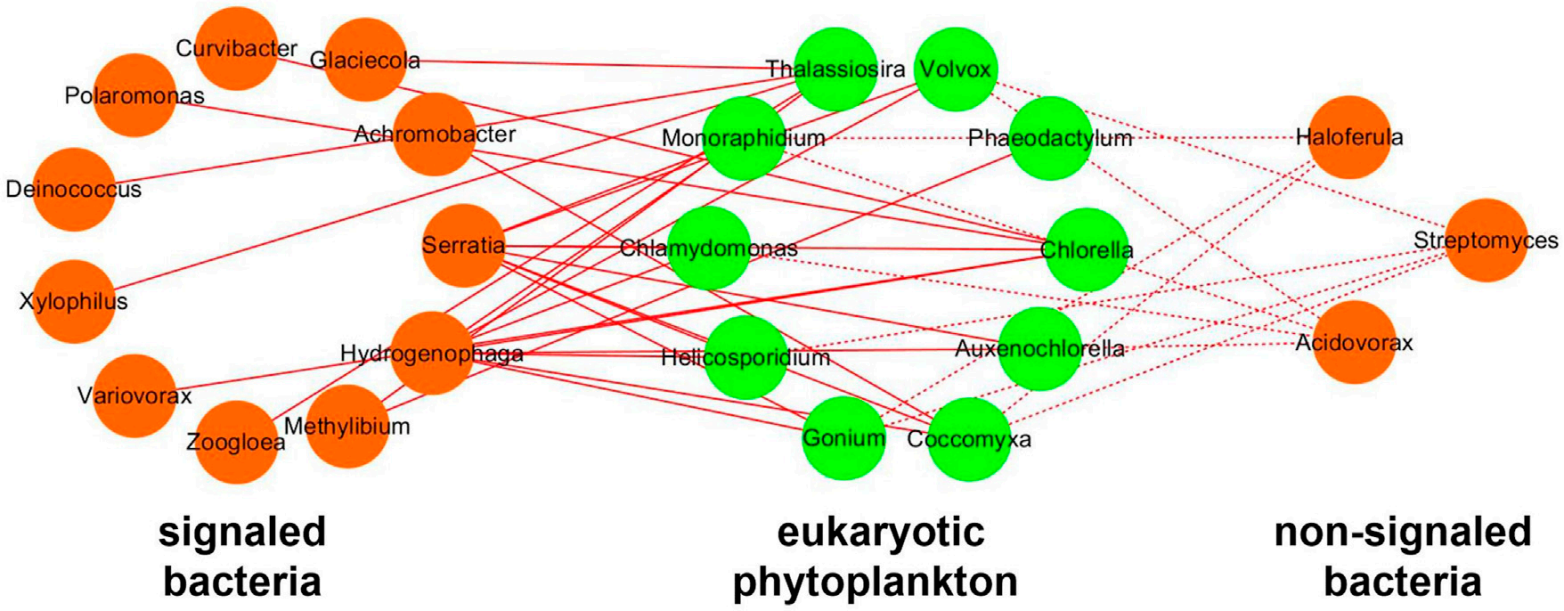
Network of positive correlation between bacteria and eukaryotic phytoplankton. Orange nodes represent bacteria, and green nodes represent phytoplankton. Solid lines represent positive correlation between signaled bacteria and phytoplankton, and dotted lines represent positive correlation between non-signaled bacteria and phytoplankton.

To figure out the relative importance of algal–bacterial signaling in the interactions, the network only using significantly positive correlations between bacteria and phytoplankton was analyzed (Figure 10). The network between signaled bacteria and phytoplankton possesses 26 pairs of significantly positive correlations, which is more than that of the network between non-signaled bacteria and phytoplankton (11 pairs). The signaling by IAA accounted for 70.27% positive correlations in the network between phytoplankton and bacteria. The positively signaled bacteria contain *Serratia*, *Hydrogenophaga*, *Methylibium*, *Zoogloea*, *Variovorax*, *Xylophilus*, *Deinococcus*, *Polaromonas*, *Curvibacter*, *Achromobacter,* and *Glaciecola*. The positively non-signaled bacteria contain *Streptomyces*, *Acidovorax,* and *Haloferula* (Figure 10).

## Discussion

### Key Role of Quorum Sensing in the Algal–Bacterial Interaction

Overall, phytoplankton prefer to positively correlate with signaled bacteria carrying IAA synthetase than non-signaled bacteria in algal reactors, which indicated that the signaling influences the algal–bacterial interaction during nitrogen removal. Particularly, the signaling between phytoplankton and bacteria accounts for 70.27% positive interaction (Figure 10), which indicates that quorum sensing might be the most crucial factor in algal–bacterial interactions in algal reactors. This finding is beneficial for understanding the mechanism of algal–bacterial interaction during water treatment using algae.

Previous research implied that algal–bacterial interaction in the phycosphere is similar with plaint–bacterial interaction in the rhizosphere (Ramanan et al., 2016; Seymour et al., 2017). The phycosphere of algal reactors represents the region surrounding a phytoplankton cell in water. Bacteria secreting IAA to the root can easily associate with plants in a soil environment (Duca et al., 2014; Philippot et al., 2013; Spaepen et al., 2007). Here, we found that signaled bacteria are more inclined to positively correlate with phytoplankton, which supports the perspective that the phycosphere is similar with the rhizosphere (Seymour et al., 2017).

### IAA Enhanced Nitrogen Removal by Promoting Biological Activity

The change tendency of IAA-related protein abundance is consistent with the nitrogen removal rate (NRR). The abundance of IAA synthetase increased during the start-up in *Chlorella* and co-culture bioreactors but decreased during the start-up in *Phormidium* bioreactors. The abundance of algal cyclin in *Chlorella* and co-culture reactors is also higher than that of the *Phormidium* reactor. Similarly, the NRR in *Chlorella* and co-culture reactors is higher than the NRR in the *Phormidium* reactor. These results imply that IAA might aggravate nitrogen removal. The bacterial IAA regulates algal cyclin after being secreted to algae, and the algal cyclin regulates cell proliferation as previously described (Amin et al., 2015; Seymour et al., 2017). Then, the biomass and activity of phytoplankton are promoted by the exchange of IAA, which might intensify the nitrogen removal efficiency in the reactors.

The main pathways of IAA synthetase during the nitrogen removal process are the IAM and IPA pathways, according to the abundance of different types of synthetase. This finding is supported by the previous research that the IAOx/IAN pathway is not the main pathway (Duca et al., 2014; Spaepen et al., 2007). Interestingly, the abundance of IAA synthetase and cyclin in different reactors implies that IAA only promotes the algal cyclin in the eukaryotic *Chlorella* reactor rather than the prokaryotic *Phormidium* reactor, which is also consistent with previous research that IAA regulates the growth of eukaryotic plants as the auxin (Duca et al., 2014; Philippot et al., 2013; Spaepen et al., 2007).

### Signaled Bacteria Carrying IAA Synthetase

The relative abundance of the total signaled bacteria in *Chlorella* and co-culture reactors is higher than that in the *Phormidium* reactor. The change trend is also similar with the NRR, which implied the signaled bacteria are also important for biological activity during nitrogen removal. *Pseudomonas*, *Hydrogenophaga,* and *Zoogloea* are the dominant signaled bacteria carrying IAA synthetase in all the reactors, and they secrete IAA into phytoplankton like rhizosphere bacteria in the soil. For instance, *Pseudomonas* provides IAA for the plant root in the soil environment (Duca et al., 2014; Spaepen et al., 2007). This is also the evidence to demonstrate that the phycosphere is similar with the rhizosphere in the soil environment which is consistent with previous research (Ramanan et al., 2016; Seymour et al., 2017). Therefore, the addition of IAA producers into algal reactors might also enhance the nitrogen removal in future studies, and it can be used in future studies, which is helpful for planning strategies of water treatment.

## Conclusion

Both *Chlorella* and co-culture reactors possessed higher nitrogen removal efficiency than the *Phormidium* reactor. The signaling dominates the algal–bacterial interaction during nitrogen removal. Quorum sensing by indole-3-acetic acid (IAA) accounts for 70.27% positive interaction between phytoplankton and bacteria. IAA was mainly synthetized depending on the indole-3-acetamide (IAM) and indole-3-pyruvic acid (IPA) pathways, and it only regulates eukaryotic phytoplankton rather than prokaryotic cyanophyta. The relative abundance of the total signaled bacteria in the whole bacterial community increased during the start-up in *Chlorella* and co-culture reactors with higher nitrogen removal rates. The signaling between phytoplankton and bacteria carrying IAA synthetase such as *Pseudomonas*, *Hydrogenophaga,* and *Zoogloea* promotes the biological activity in sequence membrane photo-bioreactors, which can enhance the nitrogen removal efficiency.

## Data Availability

The metagenomic sequences were submitted to the GenBank Sequence Read Archive (SRA) database in the National Center for Biotechnology Information (NCBI) under the accession number SRP354739.
