# Supplementary material for: Metagenomic Approaches to Explore the Quorum Sensing-Mediated Interactions Between Algae and Bacteria in Sequence Membrane Photo-Bioreactors
Source: Front Bioeng Biotechnol. 2022 Apr 5;10:851376. doi: 10.3389/fbioe.2022.851376 (PMC9036987; doi:10.3389/fbioe.2022.851376)
Supplement: Supplementary file 1 [file DataSheet1.pdf]

Supplemental information

Metagenomic sequencing and bioinformatics

Total genomic DNA was extracted using the FastDNA spin kit for soil (MP Biotechnology, CA, U.S.) according to the manufacturer's protocol. DNA purity and concentration were determined using a NanoDrop 2000 and the TBS-380 PicoGreen assay. DNA quality was checked by 1% agarose gel electrophoresis with ethidium bromide staining. The DNA samples were then used for shotgun library construction and Illumina high-throughput sequencing on the HiSeq 2500 platform at Majorbio Co., Ltd. (Shanghai, China) to generate 150 bp paired-end reads (420 bp mean insert size)

The metagenomic sequencing and all the raw reads were already processed as described previously to get the clean reads data. The raw metagenomic reads were initially trimmed by stripping the adaptor sequences and ambiguous nucleotides using SeqPrep version 1.1 based on default parameters, and the trimmed sequences were quality filtered using Sickle version 1.33 based on a minimum quality score of 20 and a minimum sequence length of 50 bp.

The metagenomic sequences were submitted to the GenBank Sequence Read Archive (SRA) database in the National Center for Biotechnology Information (NCBI) under the accession number SRP354739.

Information of datasets construction

Table S1 The entry information of IAA synthetase reads which have finally been searched in NCBI and Uniprot

IAA synthetase	Database	The entry name during the searching process
indoleacetamide hydrolase	NCBI	indole-3-acetamide hydrolase[Protein Name]<br>indoleacetamide hydrolase[Protein Name]<br>iaaH[Protein Name] AND indole-3-acetic acid<br>amidase[Protein Name] AND indole-3-acetamide<br>amidase[Protein Name] AND indole-3-acetic acid<br>amidase[Protein Name] AND indoleacetamide
	Uniprot	name: indoleacetamide hydrolase reviewed: yes<br>name: amidase AND indoleacetamide reviewed:yes<br>gene: iaaH AND indole-3-acetic acid reviewed: yes<br>name: amidase AND indole-3-acetic acid reviewed: yes<br>indole-3-acetamide hydrolase AND reviewed: yes
indoleacetaldehyde dehydrogenase	NCBI	indoleacetaldehyde dehydrogenase
		acetaldehyde dehydrogenase[Protein Name] AND indole acetaldehyde
		acetaldehyde dehydrogenase[Protein Name] AND indole-3-Acetic Acid
		aldehyde dehydrogenase[Protein Name] AND indole acetaldehyde
		aldehyde dehydrogenase[Protein Name] AND indole-3-Acetic Acid
indoleacetonitrile nitrilase	NCBI	nitrilase[Protein Name] AND indole acetonitrile
		nitrilase[Protein Name] AND indole-3-acetic acid
	Uniprot	name: nitrilase AND indole-3-acetic acid reviewed: yes

Table S2 The reads number of bacterial IAA synthetase and algal cyclin

Protein	The reads number after searching	The reads number after checking process
indoleacetamide hydrolase	3831	1008
indoleacetaldehyde dehydrogenase	40	33
indoleacetonitrile nitrilase	15	9
cyclin	444	299

Calculation of RPKM

The RPKM (Reads Per Kilobase per Million mapped reads) was calculated using the following equation:

$$Abundance(rpkm) = \sum_{1}^{n} \frac{\mathrm{N}_{mapped\ reads} \times 150/\mathrm{L}_{reference\ sequences}}{\mathrm{N}_{meta}} \times 10^6$$

where $\mathrm{N}_{\mathrm{mapped\ reads}}$ is the number of the reads in the metagenomics data that mapped to the protein sequence; $\mathrm{L}_{\mathrm{reference\ sequences}}$ is the sequence length of the corresponding reference sequence; n is the number of IAA-related reads (IAA synthetase and cyclin) belonging to the same subtype, 150 is the sequence length of the Illumina reads, and $\mathrm{N}_{\mathrm{meta}}$ is the number of the metagenomic sequence data.
